# Understanding the patient experience of heart failure with obesity and preserved ejection fraction (HFpEF): qualitative insights from patients and clinicians

**DOI:** 10.1186/s41687-026-00998-2

**Published:** 2026-01-21

**Authors:** Chisom Kanu, Tamara Al-Zubeidi, Shraddha Shinde, Gemma Al-Jassar, Jiat Ling Poon, Jordan Miller, Chris Marshall, Chloe Carmichael

**Affiliations:** 1 Eli Lilly and Company, Indianapolis, USA; 2 Clinical Outcomes Assessment, Clarivate Analytics, London, UK

**Keywords:** Heart failure with preserved ejection fraction, Obesity, Qualitative interviews, Concept elicitation, Conceptual model

## Abstract

**Background:**

Heart failure with preserved ejection fraction (HFpEF) is a heterogenous clinical syndrome. Individuals living with HFpEF and obesity display a distinct phenotype that places considerable burden on their health-related quality of life. This study explored signs, symptoms, and impacts most relevant to individuals living with HFpEF and obesity. Clinician interviews provided a clinical perspective on the patient experience.

**Methodology:**

Qualitative interviews were conducted virtually with adults in the US diagnosed with HFpEF and obesity (body mass index ≥ 30.0 kg/m2). Clinicians in the US, China, and Germany with ≥ 3 years of experience treating HFpEF were also interviewed. Patient interviews were conducted until conceptual saturation was achieved. Content analysis was conducted using Atlas.ti v9.0 software. A conceptual model (CM) of the lived experience of HFpEF and obesity based on patient and clinician feedback was developed.

**Results:**

Twenty-two patients and 6 clinicians participated. The CM demonstrated that living with HFpEF impacts many facets of life, with patient and clinician participants reporting impacts on their (or their patients’) physical functioning (e.g., walking), activities of daily living (e.g., household chores) and work. Impacts on emotional well-being (e.g., feeling anxious or worried), social lives, relationships, and sleep were also reported. Shortness of breath and fatigue were spontaneously noted as salient and bothersome symptoms by most patient participants with edema noted by most on probing; this was further corroborated by clinicians who also considered these symptoms as the most bothersome symptoms of HFpEF.

**Conclusions:**

This study highlighted the substantial burden of living with HFpEF and obesity. The resultant conceptual model highlights the key concepts that should be considered in patient-focused drug development.

**Supplementary Information:**

The online version contains supplementary material available at 10.1186/s41687-026-00998-2.

## Plain language summary

This qualitative study involved in-depth interviews with patients and health care professionals to understand the symptoms and impacts of living with heart failure with preserved ejection fraction (HFpEF) and obesity. Findings identified fatigue, shortness of breath, and swelling caused by excess fluid (edema) as core symptoms experienced by patients which subsequently impacted multiple aspects of patients’ lives including physical functioning (e.g., walking), ability to do everyday tasks (e.g., household chores), work, emotions, and sleep. The conceptual model summarizing the identified symptoms and impacts is the first to illustrate the experience of living with HFpEF and obesity and can inform future research and clinical trials for this condition.

**Supplementary Information:**

The online version contains supplementary material available at 10.1186/s41687-026-00998-2.

## Introduction

Heart failure with preserved ejection fraction (HFpEF) is a complex heterogenous clinical syndrome resulting from various pathophysiological processes. HFpEF can be defined as heart failure with an ejection fraction ≥ 50% and evidence of diastolic dysfunction and/or structural heart disease on an echocardiogram [1].The burden of heart failure in the United States (US) is rising exponentially, with the American Heart Association (AHA) projecting a 23% increase in the prevalence by 2030, from a rate of 2.42 per cent of the US population in 2012, to 2.97% by 2030. This corresponds to over eight million US adults projected to be living with heart failure by 2030 [2].

Approximately half of patients with heart failure are observed to have HFpEF [3]. Globally, the prevalence of HFpEF varies, with a review of epidemiological studies published in 2021, reporting HFpEF to affect between 1.1 and 5.5% of the general population [3, 4]. Recent evidence has also indicated an increase in the prevalence of HFpEF, attributed to both increased population aging and lifestyle related-risk factors [5, 6].

The diagnosis and treatment of HFpEF can be challenging as symptoms such as unexplained fatigue or dyspnea (shortness of breath) may appear non-specific and attributed to a number of other non-cardiac conditions [7, 8]. In addition, over 80% of patients with HFpEF are also living with overweight (Body Mass Index [BMI] > 25 kg/m^2^) or obesity (BMI > 30 kg/m^2^), which can complicate the distinction between symptoms of each condition, intensify the overall symptom burden and may contribute to diagnostic challenges [9, 10]. While obesity was previously considered to be a common comorbidity of HFpEF, evidence of the pathophysiologic mechanisms of HFpEF have now determined obesity to play a causative role in HFpEF development [11, 12]. Within the spectrum of HFpEF clinical presentation, individuals with HFpEF and obesity display a distinct phenotype where increased visceral and ectopic adiposity, and volume expansion play a role in its pathophysiology [8, 11, 13]. A comprehensive understanding of the key symptoms and impacts of these conditions could inform therapeutic approaches which are largely focused on symptomatic relief [14].

At present, there are few disease modifying agents for the treatment of HFpEF, with current therapeutic approaches largely focused on providing symptomatic relief [14]. Treating obesity and increased adiposity may improve outcomes for individuals with HFpEF and obesity [7].

Understanding the symptom experience and the concepts most relevant to individuals living with HFpEF and obesity is essential to inform clinical trial design for treatments for HFpEF and to ensure that appropriate patient-relevant outcomes are assessed. Across the literature, while one qualitative study has explored the patient experience of HFpEF [15], to the authors knowledge, the current study is the first to qualitatively explore the patient experience of those living with HFpEF and obesity in a diverse sample. While a conceptual model of the patient experience of heart failure has previously been developed by Moshkovich and colleagues (2020), given the increasing prevalence and distinct pathophysiology of HFpEF, understanding the patient perspectives from this specific clinical group to support patient-focused drug development (PFDD) is important [16]. In addition, examining the perspectives of clinicians who regularly treat patients with HFpEF provides a holistic perspective to reinforce the importance and relevance of the concepts identified by patients.

## Objectives

This non-interventional, qualitative interview study aimed to explore the experience of individuals living with HFpEF and obesity from the patient and clinician perspective to understand the concepts most relevant to patients and identify unmet treatment needs. The specific objectives of this study were primarily to understand the impact of living with HFpEF and obesity to inform the development of a conceptual model to support PFDD in this clinical group. The study also aimed to determine patients’ most bothersome symptom(s) and to examine their experience of hospitalizations and urgent visits to the emergency room (ER) due to living with HFpEF and obesity.

## Methods

### Ethical approval

The study protocol and associated documents were reviewed and approved by the Western (WIRB) Copernicus Group Institutional Review Board (WCG IRB) on 14th October 2021 (IRB Reference: 20215299).

### Recruitment and sampling strategy

Purposive sampling was used to recruit adults with a clinician-confirmed diagnosis of obesity (BMI > 30 kg/m^2^) and HFpEF [preserved left ventricular ejection fraction (LVEF) ≥ 50%] residing in the US, who were fluent in English or Spanish. Additionally, purposive sampling was used to classify the severity of heart failure via the New York Heart Association (NYHA) classification (33% of the sample with class II and 33% of the sample with class III or IV) [17]. Purposive sampling was also used to target demographic diversity in terms of sex (50% of sample male), education (33% of sample with ‘lower educational level’ defined as high school education or less), and ethnicity/race (15–25% of sample to be non-Caucasian). These targets were selected to ensure sufficient representation in non-Caucasian adults who have a disproportionate risk and adverse outcomes of heart disease, and in recognition that lower educational attainment is an established risk factor for cardiovascular disease [18–20]. Two specialist recruitment agencies, and a private clinical site in Pennsylvania were utilized for patient recruitment. The sample size for individuals living with HFpEF and obesity was determined by the principle of conceptual saturation, which is the achievement of breadth and depth of understanding defined as ‘the point at which no new information arises’ [21]. Previous research has suggested that a sample of 10–15 is usually the minimum sample size sufficient to achieve saturation of concepts in a homogenous sample [22]. Additionally, six clinicians with at least three years of experience treating individuals with HFpEF were recruited across the US, Germany and China by a specialist recruitment agency. One of the specialist recruitment agencies was used to recruit clinician participants.

Prior to participation, clinicians completed a consultant agreement form and individuals living with HFpEF and obesity completed a written informed consent form (ICF). Confirmation of diagnosis and the collection of relevant clinical details including BMI and LVEF were obtained via a clinician-completed case report form (CRF). Patient participants were asked to communicate with their clinician (or their trained clinic staff) to coordinate the completion of this document. Clinician participants received an honorarium in line with fair market value according to their geographical region ($100 (US), €100 (Germany), $150 (China); patient participants received an honorarium of $150. An additional honorarium of $50 was provided for patient participants who obtained clinical information from their physician.

### Interview procedures

This study involved open-ended interviews, lasting 30 min with clinicians and 90 min with individuals living with HFpEF and obesity. All interviews were conducted remotely using web-assisted software (Microsoft Teams) by three experienced interviewers certified in Collaborative Institutional Training Initiative (CITI) Human Subjects training. Two interviews were conducted in US-Spanish by a trained interviewer from one of the specialist recruitment agencies used during recruitment. All interviewers were trained in qualitative interview techniques and on using the study-specific interview guide to ensure a standardized approach across participants.

Semi-structured interview guides were used throughout the interviews. The guides, which were informed by PFDD guidelines [23] included open-ended concept elicitation questions and probes to explore the symptoms and impacts experienced by individuals living with HFpEF and obesity, from the perspectives of the individuals themselves and clinicians who regularly treat the condition.

To explore the most bothersome symptom(s), a series of saliency tasks and direct probing questions were used. This involved asking patient participants to identify the sign/symptom they found most bothersome out of those they had discussed. Patient participants were then asked to rank how bothersome or disturbing each identified concept was on a scale of 0 (no bother/ does not disturb) to 10 (most bothersome/ greatly disturbs) when the symptom had been at its worst. To assess most bothersome symptom(s) from the perspective of clinicians, direct questions were used.

Patient participants were additionally asked which symptom they would like a future treatment to improve, and to describe their experience of hospitalization and urgent visits to the hospital/ER due to HFpEF.

### Data analysis

Unique identifiers were used to anonymize participants data. Audio recordings from the interviews were transcribed verbatim and de-identified transcripts were analyzed using content analysis on ATLAS.ti v9.1 software. The two interviews conducted with Spanish-speaking participants were translated before analysis. The codes applied to illustrative quotes use the following denotations for patient interviews: English (EN)/Spanish (SP)-Sequence of consent-Age-Gender (e.g., EN-11-42-F) and clinician interviews: HCP-Country of residence -Sequence of interview (e.g., HCP-US-01).

Transcripts were analyzed based on semantic, qualitative, directed content analysis techniques [24] using a combination of inductive and deductive coding. The analysis used an experiential, realist approach, focusing on both patient and clinician participants’ individual perspectives and experiences. To promote methodological rigor, the direct project team met weekly to discuss the coding, sharing insights and interpretations that may have influenced the analysis. Additional meetings with wider team members not involved in the data collection were held to discuss the findings and resolve coding queries. Demographic and clinical information was descriptively summarized using Microsoft Excel.

The concepts identified were applied to inform the development of a conceptual model of HFpEF symptoms and their subsequent impact on individuals’ daily lives, well-being, and functioning.

### Conceptual saturation

Conceptual saturation was assessed for the total sample of patient participants in accordance with scientific guidance [25]. Interview transcripts were grouped into four sets in the sequential order the interviews were performed, and elicited concepts were compared between sets. The emergent codes from the last set of interviews were compared with the previous three sets to identify if any new concepts had arisen.

### Saliency graphs

Analysis of conceptual saliency enhances qualitative analysis by highlighting the extent to which a sign, symptom or impact of living with HFpEF and obesity resonates collectively across the sample based on each individual’s level of bother [26]. Saliency analysis was conducted to identify the frequency of each identified concept within patient participants’ discussions; and the significance assigned to the concepts based on their ranking of how bothersome or disturbing each concept was on a scale of 0 (no bother/does not disturb) to 10 (most bothersome/greatly disturbs). Concepts that were reported as bothersome or disturbing by > 80% of the patient participants and had an average ranking of > 5 on the 0 to 10 scale denoted higher conceptual saliency and were presented in graphical form [27].

## Results

### Individuals with HFpEF and obesity

Twenty-two participants took part in the interviews, most were completed in English (*n* = 20, 91%) and two (*n* = 2, 9%) in Spanish. The demographic and clinical characteristics of the sample are reported in Table 1. There was limited representation of male participants (*n* = 6, 27%), however all other demographic quotas were achieved, or close to the target percentage; education (32% of sample had ‘lower educational level’ defined as high school education or less), and ethnicity/race (41% of the sample was non-Caucasian). Several participants reported ≥ 1 hospitalization (*n* = 13, 59%) and ≥ 1 urgent hospital visit (*n* = 9, 41%) due to HFpEF. One female participant voluntary withdrew after providing informed consent due to illness. Data from one female participant who reported cognitive impairments during the interview (and was unable to respond to the questions independently) was excluded from the analysis.

**Table 1:** Demographic and clinical characteristics of individuals with HFpEF and obesity

Demographic and clinical characteristics	Total sample (*N* = 22)
**Demographic characteristics**	
**Age**,** years**,** Mean (Median) [Range]**	**61.5 (62.0) [42.0–77.0]**
**Gender**,** n (%)**	
Male	6 (27)
Female	16 (73)
**Race/ethnicity**,** n (%)**	
White	12 (55)
Black or African-American	5 (23)
Of Hispanic, Latino or Spanish origin	4 (18)
Prefer not to say	1 (5)
**Highest level of education completed**,** n (%)**	
Some high school/high school or equivalent	7 (32)
College or associate degree	7 (32)
Bachelor/graduate/post graduate	8 (36)
**Work status**,** n (%)**	
Retired	9 (41)
Working in paid job	7 (32)
Unemployed (not seeking employment)	3 (14)
Full-time homemaker	2 (9)
Working in unpaid/volunteer job	1 (5)
**Clinical characteristics**	
**Current BMI**,** kg/m**^**2**^, **Mean (Median) [Range]**	33.6 (31.8) [30.1–44.9]
**Most recent LVEF**,** %**,** Mean (Median) [Range]**	53.4 (53.0) [50.0–60.0]
**NYHA Classification**,** n (%)**	
II	18 (82)
III	3 (14)
IV	1 (5)
**Treatment history**,** n (%)**	
Currently on HFpEF treatment	21 (95)
Received HFpEF treatment in past	1 (5)
**Comorbidities**,** n (%)**	
Hypertension	16 (73)
Anxiety	9 (41)
Dyslipidemia	7 (32)
Depression	5 (23)
Coronary disease	4 (18)
Obstructive sleep apnea	4 (18)
T2DM	4 (18)
Angina	3 (14)
Arthritis	3 (14)
Asthma	3 (14)
Cancer	1 (5)
Stroke	1 (5)
Thyroid disease (treated)	1 (5)

Abbreviations: BMI, body mass index; HFpEF, heart failure with preserved ejection fraction; LVEF, left ventricular ejection fraction; n, number of participants; NYHA, New York Heart Association T2DM, Type 2 Diabetes Mellitus

#### Conceptual model

**Fig. 1:**
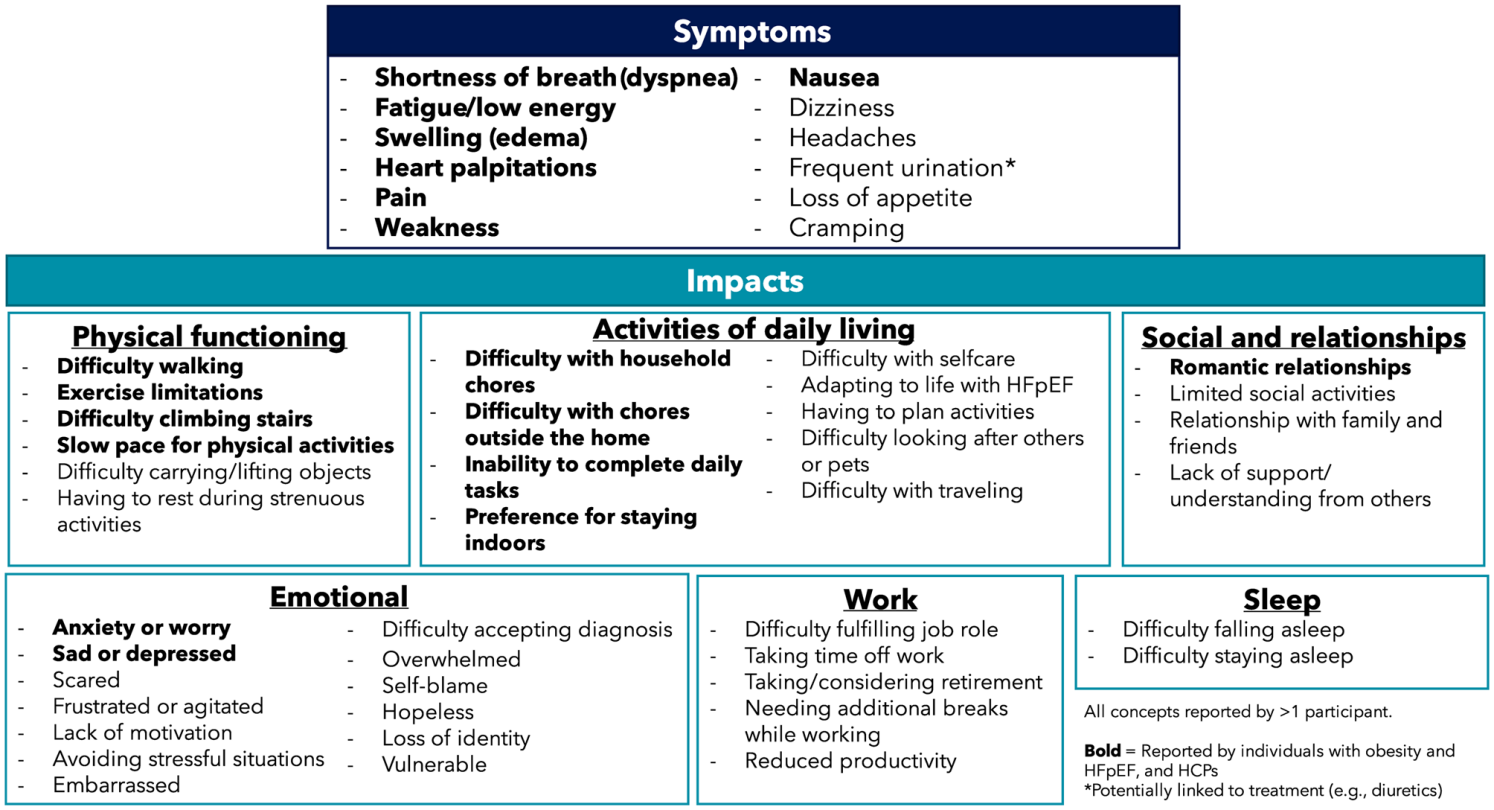
Conceptual model of the lived experience of HFpEF and obesity

Figure 1 presents a conceptual model of the experience of living with HFpEF and obesity, based on the interview findings with patient and clinician participants. The model highlights the HFpEF symptoms experienced and the impact on aspects of health-related quality of life (HRQoL) and provides insights into important concepts for patient-focused measurement strategies in clinical trials.

#### Signs and symptoms of HFpEF

All 22 participants described at least one HFpEF sign or symptom that they experienced either prior to diagnosis and/or during their experience of HFpEF. This most often included shortness of breath/dyspnea (*n* = 22, 100%), fatigue (*n* = 22, 100%) and swelling/edema (*n* = 19, 86%). Other frequently experienced symptoms included heart palpitations (*n* = 9, 41%), pain (*n* = 8, 36%), and dizziness (*n* = 8, 36%). Illustrative quotes are provided in Table 2. Descriptions and triggers for the three most commonly experienced HFpEF signs or symptoms are detailed below.

#### Shortness of breath

Participants used the terms *“shortness of breath”* or described feeling *“out of breath*” or *“winded*” when reporting this symptom. When experienced, the sensation was further described as *“suffocating”*, and *“having a hard time breathing”*, amongst other terms. Shortness of breath was often triggered by walking (*n* = 11) and over-exertion (*n* = 9) such as exercise, *“higher activity”* and *“overdoing it”*. Other triggers included completing household chores (*n* = 6), chores outside the home (*n* = 3) and the weather (*n* = 4) with cold or heat/humidity negatively impacting shortness of breath.

#### Fatigue/low energy

Participants described fatigue as tiredness, lack of stamina, and lacking in or not having enough energy. All participants (*n* = 22) described fatigue as a physical symptom. This was distinct from weakness which was described as physically feeling weak or as though their strength has declined. Some participants (*n* = 6) also experienced fatigue as a mental symptom impacting their ability to think clearly and/or complete mental tasks. Fatigue was triggered by over-exertion (*n* = 10), walking (*n* = 7), chores outside the home (*n* = 6) and/or household chores (*n* = 5).

#### Swelling or edema

Participants described edema as the presence of swelling or water/fluid retention and the feeling of edema as like having *“clubfeet”* and *“feeling like balloons”*. Most commonly, edema was experienced in the legs (*n* = 9), feet (*n* = 8), ankles (*n* = 6), and hands (*n* = 5). Triggers mentioned by one participant each (*n* = 1) included walking, eating salty food, working, heat, and positional factors such as having their hands hanging down.

**Table 2:** Summary of HFpEF signs/symptoms reported by participants

Concept	Reported by^†^			Illustrative quote(s)
	S	*P*	Total	
Shortness of breath (dyspnea)	18	4	22 (100%)	*“I get a lot of* ***shortness of breath***, *[sighs] so, if I walk way too much, you know, just, I need to sit down, I need to relax.” (EN-12-59-M)*
Fatigue/low energy	17	5	22 (100%)	*“I started, um, definitely feeling* ***even more fatigued*** *than what […] my usual was.” (EN-08-58-F)*
Swelling (edema)	3	16	19 (86%)	*“My legs will swell, swell up. Um, actually*, ***my whole-body swells***, *err, but it mainly affects me in my legs and in my face.” (EN-11-42-F)*
Heart palpitations	9	-	9 (41%)	*“Um, you know, ones that are very alarming to me would be when* ***I have palpitations****…” (EN-06-58-M)*
Pain	8	-	8 (36%)	*“Once in a while* ***I get***,*** like***,*** a sharp pain*** *on […] my, err, left side, which I was telling him about, which he later said it probably was related, but I would get this pain, like, sometime, where […] it would keep me, like, just um, immobilized, where I couldn’t move.” (EN-16-63-F)*
Dizziness	8	-	8 (36%)	*“I started noticing that, um*, ***I was having […] some dizziness***.*” (EN-01-64-F)*
Weakness	3	-	3 (14%)	*“I was actually diagnosed this year, and* ***I was feeling very weak*** *and very tired, and I just wasn’t feeling myself.” (EN-14-44-F)*
Nausea	3	-	3 (14%)	*“[…] that’s when everything is happening at once. Like*, ***I might have the nausea*** *[…] you know, sick to my stomach.” (EN-16-63-F)*
Frequent urination	2	-	2 (9%)	*“Another symptom that I get is, is the* ***frequent urination*** *[…] I’m constantly drinking water, but yet retaining some of it, and still going to the bathroom, like, 30 times a day. So, it’s exhausting and frustrating and, kind of, a lot of times, doesn’t necessarily even make sense to me.” (EN-06-58-M)*
Headaches	2	-	2 (9%)	*“Since I don’t do a lot of work and live alone, I feel exhausted when I do the chores;* ***I have headaches***.*” (SP-08-66-F)*
Loss of appetite	2	-	2 (9%)	*“I eat less […] somehow, with this condition, I don’t even feel as hungry as I used to […] sometime, it’s, err, even a turnoff, the food […]* ***I don’t feel […] my appetite is the same***.*” (EN-16-63-F)*
Cramping	2	-	2 (9%)	*“I don’t want to say body pain, but* ***cramping***, *you know, specifically in – up to the point where sometimes it feels like those muscles just can’t even go on. I, I – almost like, – at times where I just cannot continue.” (EN-06-58-M)*
Dry mouth	1	-	1 (5%)	*“…overall, just, kind of, feel terrible. You know, I’ll get* ***dry mouth*** *spells.” (EN-06-58-M)*
Migraine	1	-	1 (5%)	*“For me*, ***a migraine is not really a headache*** *so much, other than a – sort of like a pressure ache, but it was a vision impairment. I’d see a floating, um, glowing something or other than would move across the eye, and that, that was what I considered the – to be the migraine.” (EN-18-65-M)*
Nasal problems	1	-	1 (5%)	*“I have […]* ***my nasal problem***, *I guess that’s what you would call it, err, sometimes I, I wake up like I’m trying to catch my breath.” (EN-17-77-M)*
Sweating	1	-	1 (5%)	*“I sweat more during the mornings when I start my day.* ***I usually wake up sweaty*** *in the morning. I also notice that, at midnight, I start to feel sweaty too.” (SP-05-66-F)*

^†^S denotes concepts elicited **S**pontaneously; P denotes concepts elicited following **P**robed questions

#### Salient and most bothersome signs/symptoms of HFpEF

**Fig. 2:**
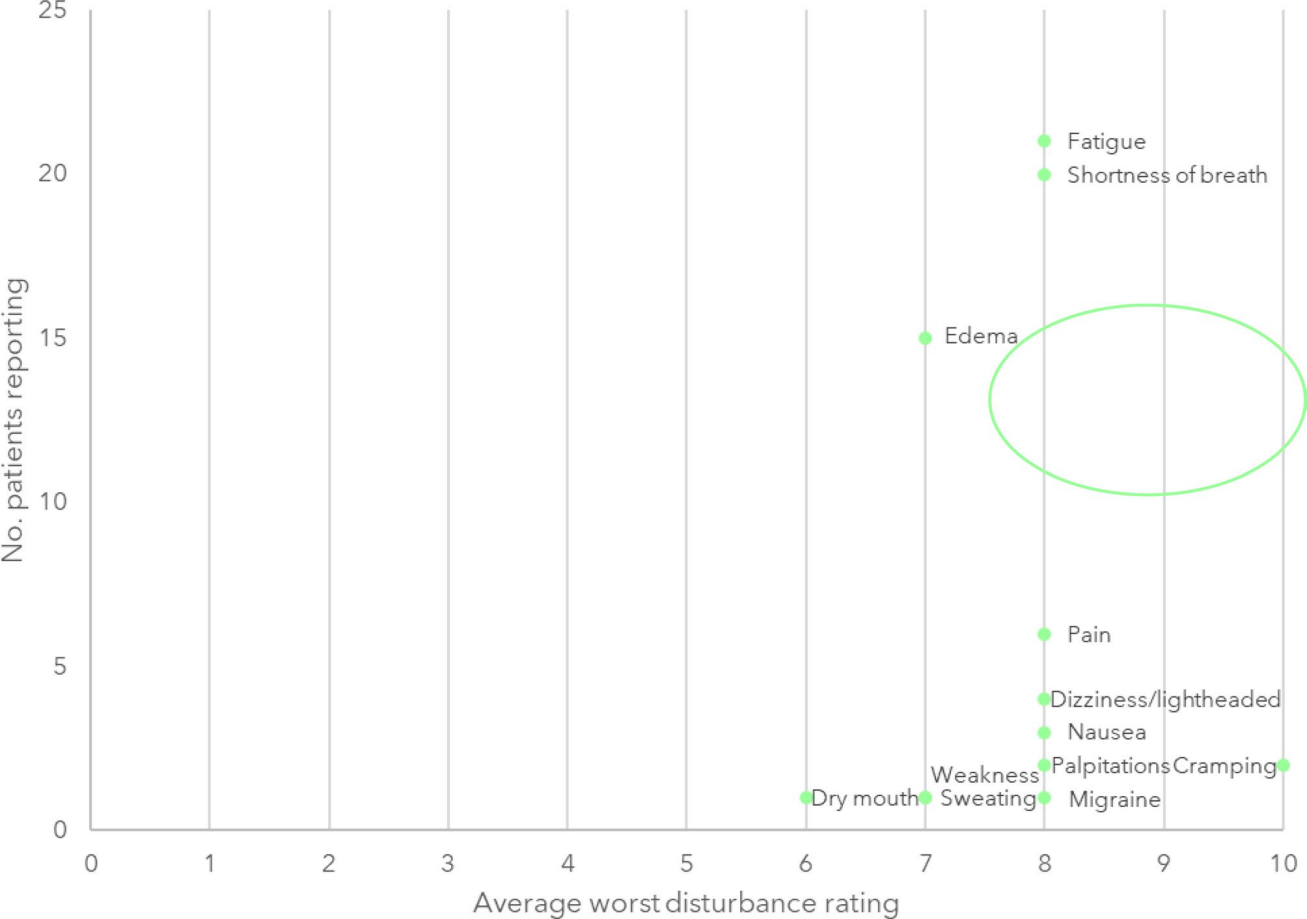
Saliency graph of HFpEF signs/symptoms at their worst. The lower ranking was included for participants who selected two choices e.g., 2 or 3

Participants’ assessment of how bothersome each sign/symptom experienced was ‘at its worst’ is summarized in Fig. 2. Fatigue and shortness of breath were considered as highly salient to the participant experience (i.e., reported by > 80% of participants with an average ranking of > 5 out of 10). Edema was also considered as highly bothersome/disturbing by a number of participants. Other symptoms including pain, dizziness/lightheadedness, nausea, heart palpitations, migraines, and cramping, were ranked as highly bothersome/disturbing by a few participants. Weakness, sweating, and dry mouth were ranked slightly less bothersome/disturbing by one participant each.

**Fig. 3:**
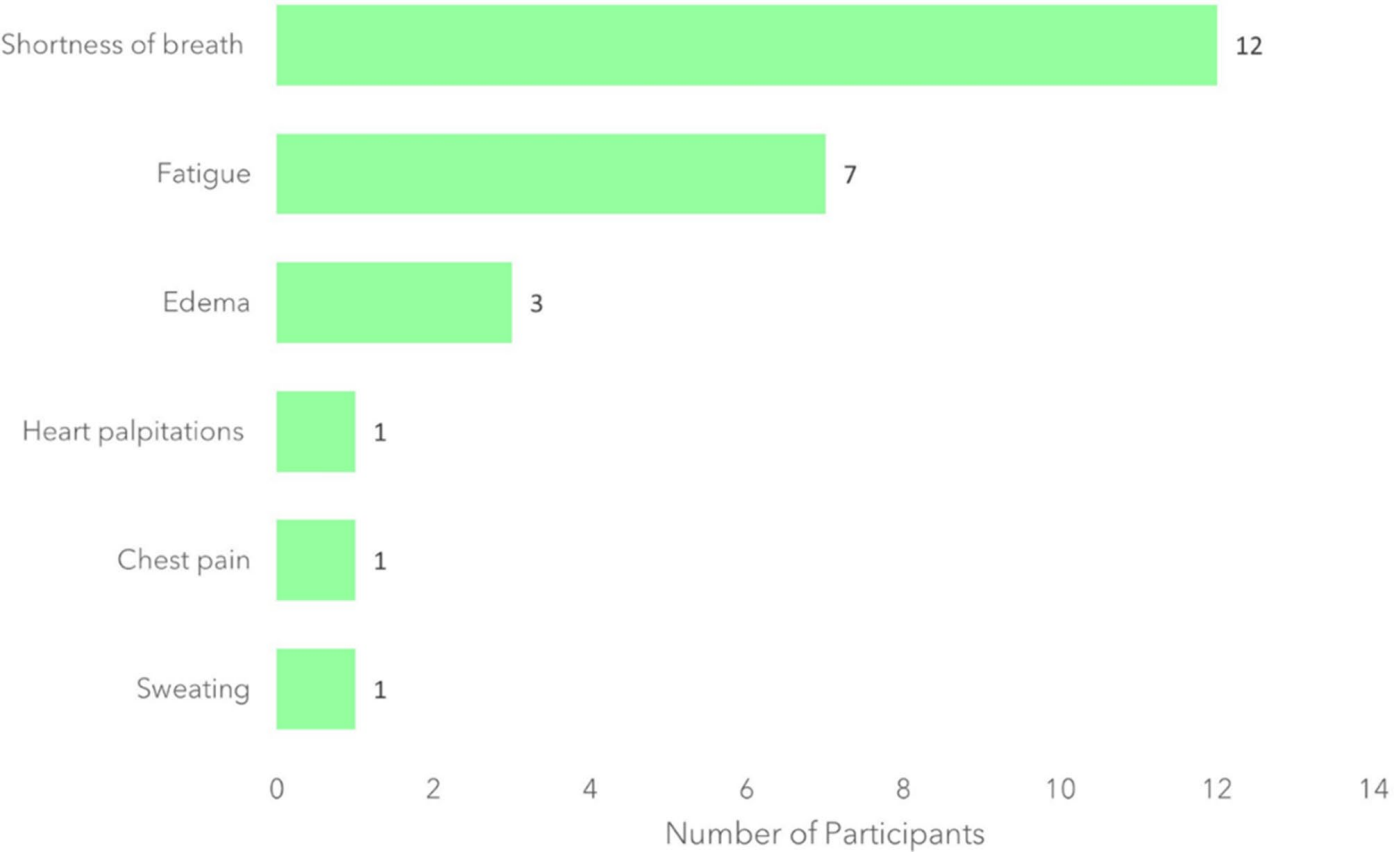
Most bothersome HFpEF signs/symptoms (*N* = 22)*. *n = 3 participants selected two most bothersome symptoms, therefore the counts total 25

All 22 participants were asked to identify the sign/symptom they found most bothersome out of those they had discussed. Figure 3 illustrates that the most frequently reported, most bothersome symptoms were shortness of breath (*n* = 12/22, 55%) and fatigue (*n* = 7/22, 32%). Five participants (*n* = 5) reported that their most bothersome symptom varied from day-to-day. Most of these participants (*n* = 4/5) indicated that their most bothersome symptom alternated between fatigue or shortness of breath.

#### Symptoms most important for a treatment to target

Twenty participants (91%) noted the symptom that they would most like a future treatment to improve. Most participants reported that shortness of breath (*n* = 11, 55%) and fatigue (*n* = 8, 40%) were the symptoms most important for a future treatment of HFpEF to target, as illustrated in the quotes below. Two participants considered the mental and physical aspects of fatigue when thinking of the symptoms they would like a future treatment to target.

just to stop the shortness of breath. Maybe I could walk a little bit longer and not be as tired. (EN-16-63-F)

for me, the fatigue, yes, because it takes over physically, it takes over mentally. (EN-02-64-F)

Other symptoms reported as important for a treatment to target included: chest pain (*n* = 2), edema (*n* = 1), “strong heartbeats” (*n* = 1), and difficulty swallowing (*n* = 1). Four participants selected two symptoms they would like treatment to improve, therefore counts total 24.

#### Impacts of HFpEF

Participants described several ways that their lives had been impacted by HFpEF, including impacts to physical functioning, activities of daily living (ADLs), social lives/relationships, emotional wellbeing, work and sleep.

#### Physical functioning

All participants (*n* = 22, 100%) reported their physical functioning to have been impacted by HFpEF. The most frequently described impact included: difficulty walking (*n* = 19, 86%) specifically in relation to distance and pace.

I can’t walk as far or as fast, err, because my breathing and stuff is not, not the same (EN-11-42-F)

Other frequently described impacts included exercise limitations (*n* = 13, 59%), difficulty climbing stairs (*n* = 13, 59%) and difficulty carrying or lifting objects (*n* = 10, 45%). These impacts were mainly attributed to shortness of breath and/or physical fatigue.

#### Activities of daily living

All participants (*n* = 22, 100%) discussed at least one way their ADLs had been impacted by HFpEF. The most frequently described impacts included: difficulty with household chores (*n* = 16, 73%) with participants reporting the need to take frequent breaks to complete their chores.

I don’t want to do anything, um, which, which means I got to rely on my daughter a lot […] I don’t want to go in the kitchen and cook a meal, ‘cause I just don’t have the breath to do it.

Participants also reported difficulty with selfcare (*n* = 14, 64%) such as bathing, dressing and grooming, difficulties completing chores outside the home (*n* = 13, 59%) such as running errands and yard work, and difficulty completing daily tasks (*n* = 11, 50%). Half of participants also more generally described impacts associated with adapting to life with HFpEF (*n* = 11, 50%).

#### Emotional wellbeing

Most participants (*n* = 20/22, 91%) reported impacts on their emotional wellbeing due to HFpEF. This most often included: anxiety/worry (*n* = 16, 73%) and feeling scared (*n* = 13, 59%), often linked to the onset and experience of symptoms such as shortness of breath and heart palpitations.

I’m very aware of my symptoms, so sometimes that’s what, kind of, brings me, like, into an anxiety. Like, “Oh no, no, no.” I try not to overthink of it – err, sorry, try not to overthink it. (EN-05-60-F)

Participants also often described feeling sad/depressed (*n* = 12, 55%) or frustrated/agitated (*n* = 9, 41%) mainly due to physical limitations imposed by their HFpEF symptoms.

#### Social lives and relationships

Seventeen participants (*n* = 17/22, 77%) discussed impacts to their social lives and/or relationships. Most often, HFpEF limited participants social activities (*n* = 12, 55%) either due to symptoms or emotional impacts.

[…] the lack of ability to just get motivated to get started to g-go somewhere […] Let’s say we have – we’re going to meet up somewhere […] You know, I may say yes, and I may be very excited about it, but when it actually comes down to it, to going, if, if I’m having not the greatest of days, just the lack of motivation, err, to absolutely even just get up and deal with it. (EN-06-58-M)

Participants also described impacts to their relationships with family and friends (*n* = 7, 32%) as they were unable to see family/ friends as often or were limited in the types of activities they could participate in.

#### Work

Twelve participants (*n* = 12/22, 55%) discussed at least one way in which HFpEF had impacted their work. Of these participants *n* = 7/12 (58%) were employed in paid or unpaid work. The most frequently described impacts included difficulty fulfilling their job role following their diagnosis (*n* = 6, 27%).

I went from a standing job, err, where I was doing a lot of walking in a warehouse, after 2019, and it’s mostly probably 80% sitting now […] I’m looking at retiring in about a year and a half, just, just be-because of the attrition, but, err, yeah, so it’s changed my job responsibilities that I’m now the desk person. (EN-03-66-F)

Other impacts included the requirement to take time off work (*n* = 6, 27%) and taking/considering early retirement (*n* = 5, 23%). Seven participants (*n* = 7/22, 32%) no longer worked for reasons unrelated to HFpEF.

#### Sleep

Eight participants (*n* = 8/22, 36%) reported their sleep had been impacted by HFpEF. This most frequently included: difficulty falling asleep (*n* = 5, 23%) due to feelings of anxiety/depression, shortness of breath or heart palpitations.

Just the, the anxiety of it – of, of that, the uncertainty, err, can get to me on certain days, sometimes trouble sleeping because of it. (EN-06-58-M)

Some participants also described difficulty staying asleep, and awoke throughout the night due to symptoms (*n* = 3, 14%) such as shortness of breath and cramping.

#### Patient perspectives on the relationship between weight and HFpEF

Although questions in relation to patients’ weight and their HFpEF symptom experience did not feature within the interview guide, almost half of participants (*n* = 10/22, 45%) made references to weight management as an important component of their HFpEF experience. Of these patients, a smaller number (3/10, 30%) explicitly noted their weight to have an impact on their HFpEF symptoms.

I think disappointed in the fact that I let my weight get so out of control and, um, I think it contributed to the heart failure (EN-01-64-F)

I know most of mine got better, um, after I changed my lifestyle of eating, and, and lost weight. It, it really made a difference. (EN-08-58-F)

#### Experience of hospitalizations and ER visits due to HFpEF

Approximately half of participants reported at least one hospitalization (required an overnight stay) (*n* = 13/22, 59%) and/or urgent visit to the ER (did not require an overnight stay) (*n* = 10/22, 45%) over the past five years, due to HFpEF. The main signs/symptoms that led participants to seek emergency care included difficulty breathing, chest pain, heart palpitations, fatigue, and edema. Less frequently reported signs/symptoms included low or high blood pressure, headache/migraine, nausea, weakness, and dizziness. The length of hospital stays varied and ranged from overnight or a few days to several months.

#### Conceptual saturation

Conceptual saturation was achieved for signs/symptoms and impacts spontaneously reported by patient participants (*N* = 22). Saturation tables are available as supplemental information.

### Clinicians

Six clinicians participated: four from the US, one from China and one from Germany. Clinicians’ demographic characteristics are reported in Table 3.

**Table 3:** Clinician demographic characteristics

Characteristic	Total sample (*N* = 6)
**Gender**,** n (%)**	
Male	5 (83)
Female	1 (17)
**Medical specialty**,** n (%)**	
Cardiologist	5 (83)
Primary care physician	1 (17)
**Type of practice**,** n (%)**	
Private hospital	3 (50)
University hospital	3 (50)
**Years treating patients with HFpEF**	
Mean (Median) [Range]	21.7 (22.5) [10–35]
**Number of HFpEF patients seen in a typical month**	
Mean (Median) [Range]	109.2 (87.5) [40–300]
**Percentage of HFpEF patients with a BMI above 30.0 kg/m**^**2**^,**%**	
Mean (Median) [Range]	42.5 (50) [5–70]

Abbreviations: BMI, body mass index; HFpEF, heart failure with preserved ejection fraction; n, number of clinicians

#### Clinical signs, symptoms and impacts of HFpEF

All clinicians described shortness of breath, fatigue, and edema (swelling) in the lower extremities as key clinical symptoms of HFpEF. Other less frequently observed symptoms included chest pain or pain on exertion, paroxysmal nocturnal dyspnea (PND), orthopnea and palpitations.

When asked which sign(s) or symptom(s) of HFpEF were most bothersome to patients, clinicians considered shortness of breath (*n* = 4/5) and fatigue (*n* = 3/5) to represent the most bothersome symptoms. One clinician identified fatigue, edema and low energy to be bothersome signs or symptoms.

Clinicians described several impacts of HFpEF on patients’ physical functioning, activities of daily living, social life, and emotional well-being (Table 4).

**Table 4:** Clinician reported impacts of HFpEF

Concept	Reported by HCPs (*N* = 6)		Illustrative quotes
Impacts on physical functioning			
Difficulty walking		5	*“They complain of vague shortness of breath or exertional shortness of breath, um, inability to walk as far as they used to, or do certain things.” (HCP-US-04)*
Reduced mobility		4	*“No, their mobility is markedly restricted, which – or can be, which is a problem.” (HCP-US-04)*
Sedentary lifestyle		3	*“Where it’s uncomfortable to move about and they’re more sedentary and it’s, kind of, a vicious cycle. It makes their, you know, err, obesity worse, like, and, um, the more they’re sitting on it, and have immobility, the more e-edema they have from their legs being dependent from sitting a long time.” (HCP-US-03)*
Difficulty exercising		2	*“I mean, you don’t, um, go to, err, err, doing sports. They just, err, limit their exercises.” (HCP-DE-06)*
Impacts on activities of daily living			
Impact on work/employment		5	*“Depends on their employment. If they’re a […] Longshoreman, yeah, they’re not going to be able to do their job. If they’re a Typist or sit at a desk all day, probably less so. It depends largely on how exertional their jobs are.” (HCP-US-04)*
Reduced ability to participate in activities		4	*“It limits their activities, they – sometimes to the point that they’re not able to perform their ADLs, they’re staying home.” (HCP-US-01)*
Difficulty completing household chores		4	*“I think it definitely impacts them. Um, you know, the-they need more help at home […]you know, like cleaning the house, err, doing laundry, preparing meals.” (HCP-US-01)*
Difficulty climbing stairs		2	*“It’s mostly exertion, walking, um, climbing the stairs, doing any kind of exercising, doing housework.” (HCP-US-04)*
Impacts on social life			
Impacts on relationships		5	*“Err, it probably, it, you know, adversely affects their performance in the home and family situation” (HCP-US-03)*
Impacts on intimacy		1	*“I mean, definitely, y-you – there could be more tension between their spouse and families. Um, it definitely can also affect, err, erection issues, err, males, um, so that’s definitely a, a concern for some.” (HCP-US-01)*
Impacts on emotional well-being			
Sadness/depression		2	*“Sometimes, I mean, it depresses them and upsets them that they’re less functional than they used to be.” (HCP-US-04)*
Anxiety		2	*“I would say that being short of breath, err, causes a lot of, um, err, psychological anx – you know, anxiety. It’s very uncomfortable to be short of breath” (HCP-US-03)*

#### Clinical perspectives on weight and HFpEF symptoms

Four clinicians (*n* = 4/6) provided insights into differences in HFpEF symptoms for those with and without obesity.

#### Shortness of breath

Most clinicians (*n* = 3/4) noted that living with obesity could exacerbate shortness of breath or make it difficult to discern HFpEF symptoms. It was also reported that shortness of breath can present earlier in individuals living with obesity. One HCP (*n* = 1/4) reported no difference in shortness of breath in HFpEF patients with and without obesity.

I’m not sure it presents differently, but it probably presents earlier and is more – makes more of an impact, because they have trouble getting around because of the obesity, as well. (HCP-US-04)

#### Fatigue

Two clinicians (*n* = 2/4) provided insights into differences in fatigue. One clinician reported no difference in fatigue in HFpEF patients with and without obesity, while one clinician described HFpEF patients with a ‘normal’ BMI experiencing less fatigue.

I would say they do differ in degree, in, in that, err, the HFpEF patients that are, err, in the normal body weight range, that have, err, you know, a normal BMI, they tend to be able to maintain activity at a higher level […] I think they’re probably less fatigued (HCP-US-03)

#### Edema

Most clinicians (*n* = 3/4) noted that living with obesity can make it difficult to discern edema. It was also reported that edema may be more likely to occur in individuals with obesity and HFpEF. One clinician (*n* = 1/4) reported no difference in edema in HFpEF patients with and without obesity.

Sometimes the edema’s difficult to establish because of their, their weight […] the symptoms may be similar, err, but sometimes more difficult for a Physician to discern. (HCP-US-02)

## Discussion

This cross-sectional, in-depth interview study highlighted the substantial impact of living with HFpEF and obesity on HRQoL. While activities related to physical functioning (e.g., walking) were most commonly reported, HFpEF also impacted activities of daily living (e.g., household chores), social life and relationships, work, emotional well-being, and sleep. With regards to symptom experiences, all patient and clinician participants reported fatigue and shortness of breath as core HFpEF symptoms. This was closely followed by edema, which was reported in almost all interviews. These core symptoms align with those reported in a qualitative study conducted with patients diagnosed with HFpEF [15]. Other prominent symptoms included heart palpations, chest pain, and pain in other body parts (arms, legs, neck). Importantly, the main symptoms reported by patient participants broadly aligned with those that clinicians had observed in their own patients. Symptoms less frequently reported by patient participants included dizziness, headaches, frequent urination, and loss of appetite.

Furthermore, fatigue and shortness of breath were noted as salient and bothersome by the majority of patient and clinician participants during the saliency and ranking tasks. Edema was also considered as highly bothersome by some patient and clinician participants. Additionally, the most frequently reported symptoms that patient participants felt were important for a future treatment to target were shortness of breath and fatigue.

While almost all patient participants were receiving treatment for HFpEF at the time of their interview, most currently experienced numerous symptoms. Around half of patients had experienced a HFpEF related hospitalization and/or an urgent visit over the past five years. The main symptoms that prompted hospitalizations or urgent visits included shortness of breath, fatigue and edema. Length of hospital stays ranged from overnight/a few days to several months.

All patient participants in this study had obesity (BMI > 30 kg/m^2^). While probes regarding weight management were not included in the interview, some patient participants spontaneously noted that weight management was an important component of living with HFpEF, with a smaller number directly reflecting on the impact of weight on their symptoms. Current evidence suggests that treating obesity can improve outcomes in patients living with HFpEF and obesity [7]. Clinicians also reported weight to influence HFpEF symptoms, namely by exacerbating shortness of breath or by making it more challenging to discern edema, a finding supported by recent research [28]. Given the potential overlap between HFpEF and obesity symptoms, individuals with a higher BMI may experience a delayed diagnosis. These insights support the use of additional sensitive diagnostic testing in patients with obesity and suspected HFpEF.

The findings of this study align with existing qualitative literature on the broader patient experience of heart failure, in which fatigue, shortness of breath, and edema were also identified as the most prevalent physiological symptoms [16]. Moreover, our findings in relation to symptom impacts also support existing literature which highlights the substantial effect of heart failure on patients’ physical functioning and daily activities, including walking and showering and the impact on patients’ social life and emotional wellbeing [16, 29, 30]. While the findings from Moshkovich et al. (2020), were applied to inform the development of a conceptual model of heart failure, the authors acknowledged that data pertaining to patients’ emotional impacts and other distal impacts were not included [16]. The current study addresses this gap and provides the first conceptual model detailing the symptoms, physical, emotional and wider distal impacts of HFpEF and obesity from the unique perspectives of those living with the condition and clinicians who manage these patients. This research expands current heart failure research by focusing on this distinct clinical group and including representation from a diverse sample. Ultimately, the concepts identified in the model should be considered as relevant and important for inclusion in patient-focused measurement strategies in clinical trials for those with HFpEF.

### Study strengths and limitations

The strengths of this study include the use of rigorous qualitative methods in line with industry guidance [31]. These methods provide depth and a breadth of understanding of the lived experience of HFpEF and obesity. The inclusion of clinician interviews further supports the clinical appropriateness of the concepts identified. The patient participant sample was also diverse in terms of age, education, and ethnicity/race which supports the generalizability of findings to patients with similar characteristics. While there was less representation of male patient participants, HFpEF is more prevalent in women [32]. The study was conducted in the US, and results may not be applicable to other countries and cultures without further investigation. Clinical diversity was also limited for breadth of BMI and NYHA class that is a marker for disease severity, therefore sub-group analysis was not possible. As this study was focused on the experiences of patients with HFpEF and obesity, the findings may not represent the perspectives of patients with HFpEF who do not also have obesity.

## Conclusion

This study highlights the substantial impact of living with HFpEF and obesity, indicating that there is an unmet treatment need for individuals living with this condition. The resultant conceptual model summarizes the key concepts that should be considered in patient-focused drug development in this population.

## Supplementary Information

Below is the link to the electronic supplementary material.

Supplementary Material 1

Supplementary Material 2

## Data Availability

The datasets generated during the study are available from the corresponding author on reasonable request.

## Abbreviations

CITI: Collaborative Institutional Training Initiative
HCP: Healthcare professional
HFpEF: Heart failure with preserved ejection fraction
HRQoL: Health-related quality of life
LVEF: Left ventricular ejection fraction
NYHA: New York Heart Association
PFDD: Patient-focused drug development
PND: Paroxysmal Nocturnal Dyspnea
T2DM: Type 2 Diabetes Mellitus
WCG-IRB: WCG Independent Review Board
