# Supplementary material for: Understanding the patient experience of heart failure with obesity and preserved ejection fraction (HFpEF): qualitative insights from patients and clinicians
Source: J Patient Rep Outcomes. 2026 Jan 21;10:25. doi: 10.1186/s41687-026-00998-2 (PMC12909683; doi:10.1186/s41687-026-00998-2)
Supplement: Supplementary file 1 — Supplementary Material 1 [file 41687_2026_998_MOESM1_ESM.docx]

**Supplemental Material**

Conceptual saturation

Conceptual saturation was assessed for signs/symptoms and impacts spontaneously reported by participants living with obesity and HFpEF (N=22).

Signs and symptoms

Figure 1 provides an overview of the saturation analysis for the signs and symptoms of HFpEF spontaneously reported by the total study sample. Conceptual saturation was achieved; almost all concepts first mentioned in Set 1 or Set 2, with only headaches mentioned spontaneously in Set 3.

Figure 1: Conceptual saturation analysis – Symptoms (N=22)

|  | **Set 1** | | | | | | **Set 2** | | | | | | **Set 3** | | | | | **Set 4** | | | | |
| --- | --- | --- | --- | --- | --- | --- | --- | --- | --- | --- | --- | --- | --- | --- | --- | --- | --- | --- | --- | --- | --- | --- |
|  | **EN-02-64-F** | **EN-03-66-F** | **EN-01-64-F** | **EN-04-69-F** | **EN-05-60-F** | **SP-04-60-F** | **EN-06-58-M** | **EN-08-58-F** | **EN-07-60-F** | **EN-10-74-F** | **EN-11-42-F** | **SP-05-66-F** | **EN-13-62-M** | **SP-08-66-F** | **EN-14-44-F** | **EN-12-59-M** | **SP-07-62-M** | **EN-15-59-F** | **EN-16-63-F** | **EN-18-65-M** | **EN-17-77-M** | **EN-19-56-F** |
| **Shortness of breath (dyspnea)** | **X** | X | X | X | X |  | X | X | X | X | X | X |  | X |  | X | X | X |  | X | X | X |
| **Fatigue/low energy** | **X** | X | X |  | X | X | X | X |  |  | X | X | X | X | X | X |  |  | X | X | X | X |
| **Dizziness** | **X** | X | X |  | X |  | X |  |  |  | X |  | X | X |  |  |  |  |  |  |  |  |
| **Nausea** | **X** |  |  |  |  |  |  |  |  |  |  |  |  | X |  |  |  |  | X |  |  |  |
| **Loss of appetite** | **X** |  |  |  |  |  |  |  |  |  |  |  |  |  |  |  |  |  | X |  |  |  |
| **Heart palpitations** |  | **X** |  |  | X |  | X | X | X | X |  |  | X | X |  |  |  |  |  | X |  |  |
| **Swelling (edema)** |  | **X** |  |  |  |  |  | X |  |  | X |  |  |  |  |  |  |  |  |  |  |  |
| **Cramping** |  |  |  |  |  |  | **X** |  |  |  |  |  |  |  |  |  |  |  |  |  |  |  |
| **Pain** |  |  |  |  |  |  | **X** |  |  | X |  | X | X | X |  |  |  |  | X |  | X | X |
| **Frequent urination** |  |  |  |  |  |  | **X** |  |  |  |  |  |  |  |  |  |  |  |  |  | X |  |
| **Weakness** |  |  |  |  |  |  |  |  |  |  | **X** | X |  |  | X |  |  |  |  |  |  |  |
| **Headaches** |  |  |  |  |  |  |  |  |  |  |  |  | **X** | X |  |  |  |  |  |  |  |  |

Key: Purple concepts were spontaneously reported in Set 1, blue in Set 2, green in Set 3 and orange in Set 4.

Impacts

Figure 2 provides an overview of the saturation analysis for the impacts associated with HFpEF that were spontaneously reported by the total study sample. Conceptual saturation was achieved; almost all concepts first mentioned in Set 1 or Set 2, with only ‘avoiding stressful situations’ mentioned spontaneously in Set 3.

Figure 2: Conceptual saturation analysis – Impacts (N=22)

|  |  | **Set 1** | | | | | | **Set 2** | | | | | | **Set 3** | | | | | **Set 4** | | | | |
| --- | --- | --- | --- | --- | --- | --- | --- | --- | --- | --- | --- | --- | --- | --- | --- | --- | --- | --- | --- | --- | --- | --- | --- |
|  |  | **EN-02-64-F** | **EN-03-66-F** | **EN-01-64-F** | **EN-04-69-F** | **EN-05-60-F** | **SP-04-60-F** | **EN-06-58-M** | **EN-08-58-F** | **EN-07-60-F** | **EN-10-74-F** | **EN-11-42-F** | **SP-05-66-F** | **EN-13-62-M** | **SP-08-66-F** | **EN-14-44-F** | **EN-12-59-M** | **SP-07-62-M** | **EN-15-59-F** | **EN-16-63-F** | **EN-18-65-M** | **EN-17-77-M** | **EN-19-56-F** |
| **Physical functioning** | **Difficulty walking** | **X** | X | X | X | X |  | X | X | X | X | X | X | X | X | X | X | X |  | X | X | X |  |
| **Physical functioning** | **Difficulty climbing stairs** | **X** | X | X | X | X |  | X |  |  |  | X | X | X |  |  |  | X |  |  | X |  |  |
| **ADL** | **Difficulty with selfcare** | **X** | X | X | X |  | X |  | X |  | X | X |  |  |  | X |  |  |  |  |  |  |  |
| **Physical functioning** | **Exercise limitations** | **X** | X |  |  | X | X |  | X | X |  | X | X | X |  | X |  | X | X |  |  |  | X |
| **ADL** | **Difficulty with chores outside the home** | **X** | X |  |  | X |  |  |  |  |  |  |  |  | X |  |  |  |  |  |  | X |  |
| **ADL** | **Having to plan activities** | **X** | X |  |  |  |  | X |  | X |  |  |  |  |  |  | X | X |  | X |  |  |  |
| **Work** | **Taking or considering retirement** | **X** | X |  |  |  |  | X |  |  |  |  |  |  |  |  |  |  |  | X |  |  |  |
| **Emotional** | **Sad or depressed** | **X** |  | X |  | X | X | X | X |  |  | X | X |  | X | X | X |  |  | X |  |  |  |
| **Emotional** | **Lack of motivation** | **X** |  | X |  |  |  | X |  | X |  | X |  | X |  |  |  |  |  |  |  |  |  |
| **Social/**  **relationships** | **Limited social activities** | **X** |  |  | X | X | X | X | X | X |  | X | X |  | X |  | X |  |  | X |  |  |  |
| **Physical functioning** | **Slow pace for physical activities** | **X** |  |  | X |  |  |  | X |  |  |  | X |  |  |  |  | X |  | X |  | X |  |
| **ADL** | **Preference for staying indoors** | **X** |  |  | X |  |  |  | X |  |  |  |  |  | X |  |  |  |  |  |  |  |  |
| **Work** | **Reduced productivity** | **X** |  |  |  |  | X |  |  |  |  |  |  |  |  |  |  |  |  |  |  |  |  |
| **Emotional** | **Anxiety or worry** |  | **X** | X |  | X |  | X | X | X |  |  | X | X | X | X | X | X | X |  | X | X | X |
| **Emotional** | **Scared** |  | **X** | X |  | X |  | X | X |  |  | X | X |  |  | X | X | X | X | X |  |  | X |
| **Work** | **Difficulty fulfilling job role** |  | **X** |  | X |  |  | X |  |  |  | X |  |  | X |  |  |  |  | X |  |  |  |
| **ADL** | **Difficulty with travel** |  | **X** |  | X |  |  |  |  |  |  |  |  |  |  |  | X |  |  |  |  |  | X |
| **ADL** | **Inability to complete daily tasks** |  | **X** |  |  |  | X | X | X |  |  | X | X | X |  | X | X | X |  |  |  | X |  |
| **ADL** | **Difficulty looking after others or pets** |  | **X** |  |  |  | X |  | X |  |  | X |  |  |  |  |  |  |  |  |  |  |  |
| **Emotional** | **Vulnerable** |  | **X** |  |  |  |  |  |  |  |  |  |  |  |  |  |  |  | X |  |  |  |  |
| **Physical functioning** | **Difficulty carrying/lifting objects** |  |  | **X** | X | X | X |  |  |  |  | X |  | X | X |  |  | X | X |  |  |  | X |
| **Emotional** | **Frustrated or agitated** |  |  | **X** |  | X |  | X | X |  |  |  | X | X | X | X |  |  |  | X |  |  |  |
| **Emotional** | **Hopeless** |  |  | **X** |  | X |  |  |  |  |  |  |  |  |  |  |  |  |  |  |  |  |  |
| **ADL** | **Adapting life to HFpEF** |  |  | **X** |  |  | X | X |  | X |  | X | X |  | X | X | X | X | X |  |  |  |  |
| **Work** | **Taking time off work** |  |  | **X** |  |  |  | X | X |  |  |  |  |  |  |  |  |  |  |  |  |  |  |
| **ADL** | **Difficulty with household chores** |  |  |  | **X** | X |  |  | X | X |  |  |  | X |  | X | X |  |  |  |  |  |  |
| **Social/**  **relationships** | **Lack of support/ understanding from others** |  |  |  |  |  | **X** |  | X |  | X |  |  |  |  |  |  |  |  |  |  |  |  |
| **Emotional** | **Self-blame** |  |  |  |  |  | **X** |  | X |  |  |  |  |  |  | X |  |  |  |  |  |  |  |
| **Emotional** | **Embarrassed** |  |  |  |  |  |  | **X** | X | X |  |  |  |  |  |  |  |  |  |  |  |  |  |
| **Sleep** | **Difficulty falling asleep** |  |  |  |  |  |  | **X** | X |  |  | X |  | X | X |  |  |  |  |  | X |  |  |
| **Sleep** | **Difficulty staying asleep** |  |  |  |  |  |  | **X** | X |  |  |  |  |  |  |  |  |  |  |  |  |  |  |
| **Emotional** | **Loss of identity** |  |  |  |  |  |  | **X** |  |  |  | X |  |  |  |  |  |  |  |  |  |  |  |
| **Social/**  **relationships** | **Relationship with family and friends** |  |  |  |  |  |  | **X** |  |  |  |  | X |  |  |  |  | X |  |  |  |  | X |
| **Social/**  **relationships** | **Romantic relationships** |  |  |  |  |  |  | **X** |  |  |  |  | X |  |  |  |  |  |  |  |  |  |  |
| **Emotional** | **Overwhelmed** |  |  |  |  |  |  | **X** |  |  |  |  |  |  | X |  | X |  |  |  |  |  |  |
| **Work** | **Needing additional breaks while working** |  |  |  |  |  |  | **X** |  |  |  |  |  |  | X |  |  |  |  |  |  |  |  |
| **Physical functioning** | **Having to rest during strenuous activities** |  |  |  |  |  |  |  | **X** |  |  |  |  | X |  | X | X |  |  | X |  |  |  |
| **Emotional** | **Difficulty accepting diagnosis** |  |  |  |  |  |  |  | **X** |  |  |  |  | X |  | X |  |  |  |  |  |  |  |
| **Emotional** | **Avoiding stressful situations** |  |  |  |  |  |  |  |  |  |  |  |  | **X** |  |  |  | X | X |  | X |  |  |

Key: Purple concepts were spontaneously reported in Set 1, blue in Set 2, green in Set 3 and orange in Set 4
