# Supplementary material for: Understanding the patient experience of heart failure with obesity and preserved ejection fraction (HFpEF): qualitative insights from patients and clinicians
Source: J Patient Rep Outcomes. 2026 Jan 21;10:25. doi: 10.1186/s41687-026-00998-2 (PMC12909683; doi:10.1186/s41687-026-00998-2)
Supplement: Supplementary file 2 — Supplementary Material 2 [file 41687_2026_998_MOESM2_ESM.docx]

| **Code** | **Additional detail** |
| --- | --- |
| **Impact codes** | |
| Ix-ADL-Adapting life to HFpEF | **Domain**: activities of daily living  Codes applied when impacts were described to impact activities of daily living and daily tasks, including chores in and outside the house, self-care, ability to travel and leave the home due to HFpEF. |
| Ix-ADL-Chores inside |  |
| Ix-ADL-Chores outside |  |
| Ix-ADL-Completing daily tasks |  |
| Ix-ADL-Independence |  |
| Ix-ADL-Looking after others |  |
| Ix-ADL-Planning |  |
| Ix-ADL-Selfcare |  |
| Ix-ADL-Stay at home |  |
| Ix-ADL-Travel |  |
| Ix-Cognition-Mental capacity | **Domain**: cognition  Codes applied when impacts were described to cognition due to HFpEF. |
| Ix-Emo-Anxiety/Worry | **Domain**: emotional wellbeing  Codes applied when impacts were described to emotional wellbeing due to HFpEF. |
| Ix-Emo-Embarrassed |  |
| Ix-Emo-Frustrated/Agitated |  |
| Ix-Emo-Hopeless |  |
| Ix-Emo-Loss of identity |  |
| Ix-Emo-Motivation |  |
| Ix-Emo-Not accepting HFpEF |  |
| Ix-Emo-Overwhelmed |  |
| Ix-Emo-Scared |  |
| Ix-Emo-Self blame |  |
| Ix-Emo-Stressful |  |
| Ix-Emo-Vulnerable |  |
| Ix-Financial | **Domain:** financial  Codes applied when impacts were described to participants finances due to HFpEF. |
| Ix-Food-Diet | **Domain:** food  Codes applied when impacts were described in relation to participants food intake or choices due to HFpEF. |
| Ix-Food-Diet-Amount of food |  |
| Ix-Food-Diet-Difficulty with changes |  |
| Ix-Food-Diet-Family changed diet |  |
| Ix-Food-Diet-Increase water intake |  |
| Ix-Food-Diet-Timing of food |  |
| Ix-Food-Diet-Timing of food-Uncomfortable |  |
| Ix-Phys-Carrying/Lifting items | **Domain**: physical functioning  Codes applied when impacts were described in relation to participants physical functioning due to HFpEF. |
| Ix-Phys-Exercise |  |
| Ix-Phys-Slow pace |  |
| Ix-Phys-Stairs |  |
| Ix-Phys-Strenuous activities |  |
| Ix-Phys-Walking |  |
| Ix-Sleep-Difficulty sleeping | **Domain:** sleep  Codes applied when impacts were described in relation to participants sleep due to HFpEF. |
| Ix-Sleep-Sleeping longer |  |
| Ix-Sleep-Waking due to symptoms |  |
| Ix-Soc-Family/Friends | **Domain**: social life and relationships  Codes applied when impacts were described in relation to participants social life and relationships due to HFpEF. |
| Ix-Soc-Intimacy |  |
| Ix-Soc-Lack of support/understanding |  |
| Ix-Soc-Limited activities |  |
| Ix-Soc-Romantic |  |
| Ix-Soc-Uncomfortable in crowds |  |
| Ix-Work-Anxiety | **Domain:** work  Codes applied to any work-associated impacts, due to HFpEF. |
| Ix-Work-Job role |  |
| Ix-Work-Not relevant (Not HFpEF) |  |
| Ix-Work-Not relevant-Does not work |  |
| Ix-Work-Not relevant-Not working due to other conditions |  |
| Ix-Work-Productivity |  |
| Ix-Work-Retirement |  |
| Ix-Work-Taking breaks |  |
| Ix-Work-Time away from work |  |
| Ix-Work-Unable to work |  |
| **Symptom codes** | |
| Sx-Appetite loss | **Symptoms**  Symptoms described by participants including symptom description, location and triggers where appropriate. |
| Sx-Asymptomatic |  |
| Sx-Cramping |  |
| Sx-Dizziness |  |
| Sx-Dizziness-Trigger-Chores inside |  |
| Sx-Dizziness-Trigger-Heat |  |
| Sx-Dizziness-Trigger-Movement |  |
| Sx-Dizziness-Trigger-Stairs |  |
| Sx-Dizziness-Trigger-Walking |  |
| Sx-Dizziness-Trigger-Work |  |
| Sx-Dry mouth |  |
| Sx-Edema |  |
| Sx-Edema-Area-Ankles |  |
| Sx-Edema-Area-Arms |  |
| Sx-Edema-Area-Face |  |
| Sx-Edema-Area-Feet |  |
| Sx-Edema-Area-Hands |  |
| Sx-Edema-Area-Hips |  |
| Sx-Edema-Area-Legs |  |
| Sx-Edema-Area-Thighs |  |
| Sx-Edema-Description |  |
| Sx-Edema-Trigger-Body position |  |
| Sx-Edema-Trigger-Eating |  |
| Sx-Edema-Trigger-Heat |  |
| Sx-Edema-Trigger-Salty food |  |
| Sx-Edema-Trigger-Walking |  |
| Sx-Edema-Trigger-Working |  |
| Sx-Fatigue |  |
| Sx-Fatigue-Desc-Mental |  |
| Sx-Fatigue-Desc-Physical |  |
| Sx-Fatigue-Trigger-Activities |  |
| Sx-Fatigue-Trigger-Anxiety |  |
| Sx-Fatigue-Trigger-Chores inside |  |
| Sx-Fatigue-Trigger-Chores outside |  |
| Sx-Fatigue-Trigger-Heat/humidity |  |
| Sx-Fatigue-Trigger-Lack of sleep |  |
| Sx-Fatigue-Trigger-Over exertion |  |
| Sx-Fatigue-Trigger-Stairs |  |
| Sx-Fatigue-Trigger-Unhealthy food |  |
| Sx-Fatigue-Trigger-Varies |  |
| Sx-Fatigue-Trigger-Walking |  |
| Sx-Headaches |  |
| Sx-Headaches-Triggers |  |
| Sx-Heart palpitations |  |
| Sx-Migraine |  |
| Sx-Nasal problems |  |
| Sx-Nausea |  |
| Sx-Nausea-Trigger-Mobility |  |
| Sx-Pain |  |
| Sx-Pain-Description |  |
| Sx-Pain-Location-Arms |  |
| Sx-Pain-Location-Chest |  |
| Sx-Pain-Location-Left side |  |
| Sx-Pain-Location-Legs |  |
| Sx-Pain-Location-Neck |  |
| Sx-Pain-Trigger-Activities |  |
| Sx-Pain-Trigger-Edema |  |
| Sx-Pain-Trigger-Moving abruptly |  |
| Sx-Shortness of breath |  |
| Sx-Shortness of breath-Description |  |
| Sx-Shortness of breath-Position-getting up from chair |  |
| Sx-Shortness of breath-Position-Lying flat |  |
| Sx-Shortness of breath-Position-Sitting |  |
| Sx-Shortness of breath-Position-Standing |  |
| Sx-Shortness of breath-Trigger-Activity |  |
| Sx-Shortness of breath-Trigger-Agitated |  |
| Sx-Shortness of breath-Trigger-Anxiety |  |
| Sx-Shortness of breath-Trigger-Carrying |  |
| Sx-Shortness of breath-Trigger-Chores inside |  |
| Sx-Shortness of breath-Trigger-Chores outside |  |
| Sx-Shortness of breath-Trigger-Drinks |  |
| Sx-Shortness of breath-Trigger-Fatigue |  |
| Sx-Shortness of breath-Trigger-Fluid |  |
| Sx-Shortness of breath-Trigger-Heart Attack |  |
| Sx-Shortness of breath-Trigger-Hiking |  |
| Sx-Shortness of breath-Trigger-Hurrying |  |
| Sx-Shortness of breath-Trigger-Not enough food |  |
| Sx-Shortness of breath-Trigger-Over exertion |  |
| Sx-Shortness of breath-Trigger-Shortness of breath |  |
| Sx-Shortness of breath-Trigger-Stairs |  |
| Sx-Shortness of breath-Trigger-Walking |  |
| Sx-Shortness of breath-Trigger-Weather |  |
| Sx-Strong heart rate |  |
| Sx-Strong heart rate-Triggers-Poor sleep |  |
| Sx-Sweating |  |
| Sx-Urination problems |  |
| Sx-Weakness |  |
| Sx-Weakness-Description |  |
| Sx-Impacted/improved by weight loss | Perspectives on weight management and the impact of weight loss on HFpEF symptoms. |
| Coping/Disease management-Weight management/loss |  |
| **Saliency task** | |
| Saliency task-Cramping-At its worst-10 | Scores provided by participants during the saliency task in response to the question ‘On a scale of 0 to 10, where 0 means that this symptom was of no bother to you and 10 means that the symptom was greatly bothersome, how bothersome was [symptom] at its worse? |
| Saliency task-Cramps-At its worst-9 |  |
| Saliency task-Dizziness/lightheaded-At its worst-7 or 8 |  |
| Saliency task-Dizziness/lightheaded-At its worst-8 |  |
| Saliency task-Dizziness/lightheaded-At its worst-8 or 9 |  |
| Saliency task- Dizziness/lightheaded-At its worst-9 |  |
| Saliency task-Dry mouth-At its worst-6 |  |
| Saliency task-Edema-At its worst- 8 or 9 |  |
| Saliency task-Edema-At its worst-10 |  |
| Saliency task-Edema-At its worst-3 |  |
| Saliency task-Edema-At its worst-4/5 |  |
| Saliency task-Edema-At its worst-5 |  |
| Saliency task-Edema-At its worst-5 or 6 |  |
| Saliency task-Edema-At its worst-7 |  |
| Saliency task-Edema-At its worst-9 |  |
| Saliency task-Edema-At its worst-9/10 |  |
| Saliency task-Fatigue-At its worst-10 |  |
| Saliency task-Fatigue-At its worst-4 |  |
| Saliency task-Fatigue-At its worst-5 |  |
| Saliency task-Fatigue-At its worst-5/6 |  |
| Saliency task-Fatigue-At its worst-7 |  |
| Saliency task-Fatigue-At its worst-8 |  |
| Saliency task-Fatigue-At its worst-8/9 |  |
| Saliency task-Fatigue-At its worst-9 |  |
| Saliency task-Migraine-At its worst-8 |  |
| Saliency task-Nausea-At its worst-7 |  |
| Saliency task-Nausea-At its worst-8 |  |
| Saliency task-Nausea-At its worst-8/9 |  |
| Saliency task-Pain-At its worst-10 |  |
| Saliency task-Pain-At its worst-7 |  |
| Saliency task-Pain-At its worst-8 |  |
| Saliency task-Palpitations -At its worst-7 |  |
| Saliency task-Palpitations-At its worst-9 |  |
| Saliency task-Shortness of breath-At its worst-10 |  |
| Saliency task-Shortness of breath-At its worst-4 |  |
| Saliency task-Shortness of breath-At its worst-7 |  |
| Saliency task-Shortness of breath-At its worst-7/8 |  |
| Saliency task-Shortness of breath-At its worst-8 |  |
| Saliency task-Shortness of breath-At its worst-8/9 |  |
| Saliency task-Shortness of breath-At its worst-9 |  |
| Saliency task-Sweating-At its worst-7 |  |
| Saliency task-Weakness-At its worst-7 |  |
| **Most bothersome symptoms** | |
| Sx-Most bothersome-Changes | Symptoms of HFpEF patients reported were the most bothersome symptoms. |
| Sx-Most bothersome-Chest pain |  |
| Sx-Most bothersome-Edema |  |
| Sx-Most bothersome-Fatigue |  |
| Sx-Most bothersome-Palpitations |  |
| Sx-Most bothersome-Shortness of breath |  |
| Sx-Most bothersome-Sweating |  |
| **Symptoms most important to improve** | |
| Sx-Most like to improve-Edema | Symptoms patients would most like to improve about their HFpEF. |
| Sx-Most like to improve-Fatigue |  |
| Sx-Most like to improve-Not completed |  |
| Sx-Most like to improve-Pain |  |
| Sx-Most like to improve-Shortness of breath |  |
| Sx-Most like to improve-Strong heartbeats |  |
| Sx-Most like to improve-Swallowing |  |
| **Experience of Hospitalisations and Urgent Visits** | |
| TL-Hospitalisation-None | No experience of hospitalisation or urgent visits due to HFpEF. |
| TL-Urgent visits-None |  |
| TL-Hospitalisation-Medication/Treatment | Purpose or reason for hospitalisation or urgent visit reported by participants. |
| TL-Hospitalisation-Tests |  |
| TL-Hosp-Reason-Angioplasty |  |
| TL-Hosp-Reason-Stent |  |
| TL-Hosp-Reason-Valve replacement |  |
| TL-Urgent visits-Medication/Treatment |  |
| TL-Urgent visits-Reason-Tests completed |  |
| TL-Urgent visits-Symptoms |  |
| TL-Urgent visits-Tests |  |
| TL-Hosp-Length of stay | Lenth of time participants spent in hospital. |
| TL-Urgent visits-Length of stay |  |
| TL-Number of hosps | Number of urgent visits of hospitalisations recalled by participants. |
| TL-Number of UVs |  |
| TL-Sx-Dizziness | Symptoms that led to participants requiring an urgent visit or hospitalisation. |
| TL-Sx-Edema |  |
| TL-Sx-High or Low blood pressure |  |
| TL-Sx-Fatigue |  |
| TL-Sx-Heart palpitations |  |
| TL-Sx-Migraine |  |
| TL-Sx-Nausea |  |
| TL-Sx-Pain |  |
| TL-Sx-Shortness of breath |  |
| TL-Sx-Weakness -Trouble walking |  |
